# Critical Artifacts Improve Reproducibility of Protein–Ligand Binding Affinity Prediction Models on CASF-2016

**DOI:** 10.1021/acs.jcim.6c01192

**Published:** 2026-06-24

**Authors:** Joelle N. Eaves, Angeline A. Needs, Daniel R. Woldring

**Affiliations:** † Department of Chemical Engineering and Materials Science, 3078 Michigan State University, East Lansing, Michigan 48824, United States; ‡ Institute for Quantitative Health Science and Engineering, 3078 Michigan State University, East Lansing, Michigan 48824, United States

## Abstract

Protein–ligand binding affinity prediction (PLBAP) models are routinely benchmarked on the CASF-2016 data set with Pearson correlation coefficient (PCC) as a common measure of scoring power. Published PCC values are frequently reused as baselines for cross-study comparisons. This practice implicitly assumes that published pipelines remain runnable and that reported metrics can be independently verified. To examine this assumption, we conducted a systematic reproducibility audit of 50 PLBAP models published between 2021 and 2024 that reported CASF-2016 scoring power. For each model, we attempted to reproduce the authors’ CASF-2016 inference using only publicly available code, documentation, and pretrained weights. To scaffold this audit and to offer a reusable resource for the community, we introduce a minimal five-item reproducibility checklist for PLBAP pipelines, organized around the artifacts a researcher requires to independently rerun inference: (1) a license; (2) preprocessing and featurization, (3) training, and (4) inference code; and (5) pretrained model weights. We find that only 17/50 pipelines satisfied all checklist items to be consistently runnable. Of those 17 runnable models, only nine were statistically reproducible (53% of models). We propose the checklist as a lightweight community standard for future PLBAP releases, document common gaps, and highlight practices that most reliably enabled independent reproduction.

## Graphical abstract

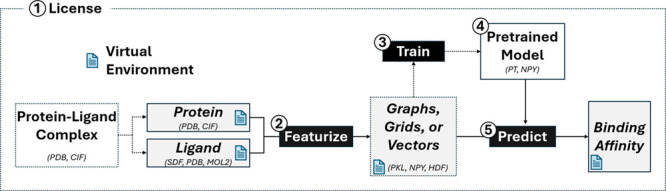

## Introduction

Reproducibility is critical to scientific progress and credibility. It enables cumulative methodological improvements, independent verification, and cross-study comparisons.^1^ In the context of machine learning, practical reproducibility becomes more difficult as models grow more complex and software stacks more fragile.^2,3^ Protein–ligand binding affinity prediction models (PLBAP) exemplify this trend; modern pipelines integrate heterogeneous preprocessing steps, specialized featurization protocols, deep learning architectures, and large pretrained models.

The majority of state-of-the-art PLBAP models report performance on the CASF-2016 benchmark,^4^ which is a set of 285 protein–ligand pairs with high-resolution crystal complex structures and verified experimental binding affinities. Model performance (i.e., scoring power) is typically summarized by the Pearson correlation coefficient (PCC). CASF-2016 has become the reference point for cross-study comparisons to support claims of improved predictive accuracy.^5–9^ In many publications, previously reported CASF-2016 metrics are directly reused as baselines for comparison without rerunning the models to verify the originally reported performance. This implicitly assumes that the original pipelines remain runnable and that their results are readily verifiable.

Prior work has noted reproducibility challenges in machine learning broadly^3,2,10^ and in computational drug discovery specifically,^11,12^ but a systematic, quantitative audit of reproducibility across PLBAP models evaluated on a single benchmark has not been reported. Such an audit is timely. The field has accelerated rapidly, with dozens of architectures, from voxel-based CNNs to equivariant graph networks, reporting CASF-2016 improvements in recent years.^13^ Understanding which pipelines can actually be rerun is intuitively useful. More importantly, though, we aim to understand what distinguishes reproducible pipelines from those that cannot be run or reproduced.

Rather than framing a critique of individual models, our goals are (1) to identify and applaud the practices that enable independent reproduction and (2) to translate these observations into actionable, forward-looking guidance. To that end, we introduce a five-item reproducibility checklist (Figure 1, Table 1) of artifacts required for an independent researcher to rerun CASF-2016 inference end-to-end. We then apply this checklist to 50 PLBAP models published between 2021 and 2024. We attempt to reproduce each model, then use the outcomes to validate the checklist as a minimum requirement for practical reproducibility. Our hope is that this checklist becomes a lightweight standard which enables future PLBAP releases to be more reliable.

**Figure 1:**
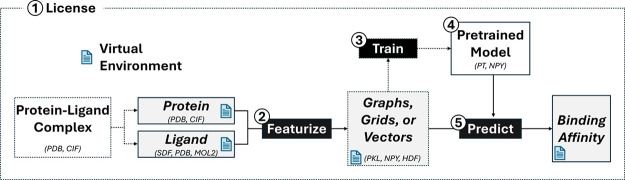
Common protein–ligand binding affinity prediction (PLBAP) pipeline steps, highlighting the five reproducibility-critical artifacts with numbered circles. Also indicated with blue document icons are additional useful materials that, while not *necessary* for reproduction, substantially improve the ease and longevity of reproducibility. These include the exact environment used to obtain the originally reported results (e.g., a conda environment file or container), example input and intermediate files, and the per-complex predictions for the entire test data set.

**Table 1:**
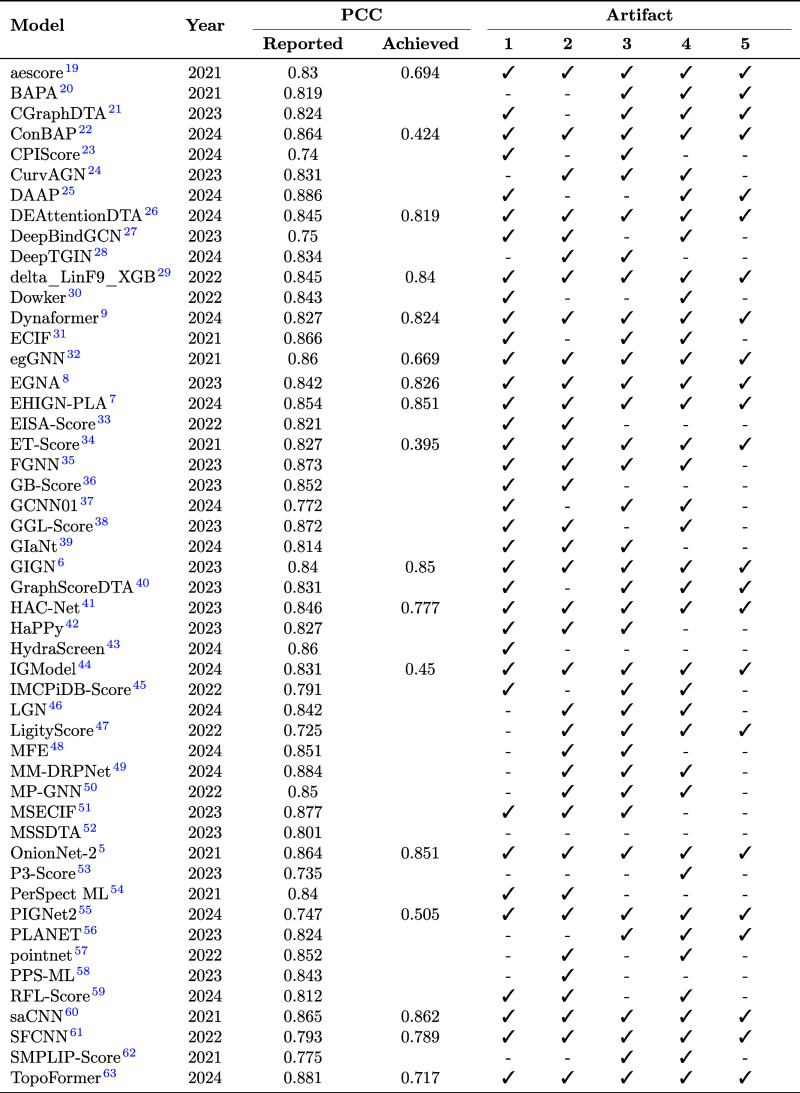
Reproducibility-Critical Artifact Availability for 50 Audited PLBAP Models, Organized by Publication Year^a^,^b^

a Artifact columns numbered as in Figure 1: (1) license; (2) preprocessing and featurization code; (3) training code; (4) inference code; (5) pretrained weights.

b Column numbers correspond to the artifact callouts in Figure 1. 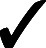 provided; - not provided.

To support adoption of the checklist and to provide a reusable resource for the community, we make all study forks, environment files, per-complex predictions, and reproduction metadata publicly available. The 17 documented study forks hosted on GitHub represent an independently useful resource beyond this manuscript: each contains commit-level documentation of every modification made to achieve a runnable state, a tested conda environment specification, and SLURM submission scripts compatible with standard HPC infrastructure.

## Results and Discussion

### Checklist of Reproducibility-Critical Artifacts

Inspired by the success of checklists in safety-critical fields such as aviation and healthcare,^14–16^ the computational research community has begun adopting similar frameworks to encourage best practices for reproducibility. A report from the 2019 Neural Information Processing Systems (NeurIPS) conference reproducibility program found that a 17-point checklist provided to authors and reviewers supported key practices such as data sharing, code availability, and compute definitions.^10^ Similar themes were discussed in a 2021 Nature publication that described a tiered scheme for evaluating reproducibility in the life sciences.^17^ Further emphasizing the application of these principles in specialized domains, a 2023 publication identified five core elements for reproducibility in bioinformatics: literate programming, code availability, defined compute environments, data sharing, and adequate documentation.^18,20,21,23–25^ Building on these precedents, we propose a practical reproducibility checklist to provide rigid definitions specific to PLBAP pipelines.

A practical reproducibility checklist for PLBAP pipelines should satisfy two criteria: it must be (i) *minimal*, containing only artifacts whose absence creates a genuine hard barrier to independent reproduction, and (ii) *actionable*, specifying artifacts that authors can realistically provide at the time of publication.^27–31,33,35–37^ We identify five such artifacts that mirror the sequential stages of CASF-2016 inference: (1) license, (2) preprocessing and featurization code, (3) training code, (4) inference code, and (5) pretrained weights (Figure 1). Beyond these five *essential* items, several supplementary artifacts can substantially improve reproducibility and we highly encourage their release alongside the five primary checklist items. These are a defined compute environment, intermediate featurized data files, documented train/test splits and random seeds, and per-complex prediction results for the test set. While these artifacts dramatically improve convenience of running and reproduction odds, they are not included in the primary checklist because they are either (a) not entirely necessary for rerunning—in the case of environment files and example data—or (b) are already standard practice to include in main texts (e.g., train/test splits). We discuss each in the Future Proofing section and strongly recommend them as best practices for PLBAP model authors. The five-item checklist here is merely our suggestion for a minimum requirement.

### Artifact Availability Across Modern Pipelines

We applied the checklist to 50 PLBAP models published between 2021 and 2024.^38–40,42,43,45–54^ Table 1 summarizes artifact availability for each model alongside its publication year. The table reveals substantial heterogeneity: some pipelines satisfied all checklist items, while others were missing all.

#### License Availability

Critical to reuse of any intellectual property is a suitable license. A license is a document that explicitly defines how others can use, modify, or distribute materials^56–62^ . For data and code intended to be open source, there are common licenses, such as the MIT license,^64^ GNU GPLv3,^65^ and the Apache-2.0 license,^66^ which impart the necessary protections and permissions and are easily added to code repositories. Without a license, default copyright exclusivity applies and external users have no rights to use or modify the published software, inhibiting others from reproducing the results or using the software as intended.^67^ While fair use exceptions provide some leniency, particularly for noncommercial or academic research, using unlicensed data or code is risky.^68,69^

Initially, 21 of the 50 PLBAP models evaluated (42%) had no license and were thus unusable. For each of these models, if a GitHub repository was available, a public issue was posted requesting a license be uploaded. Similarly, if the repository was hosted on GitLab, the corresponding author was emailed with a license upload request. Pleasantly, after given a minimum of 3 weeks to respond, license availability did significantly improve. At the time of writing, 7 licenses had been added, leaving only 14 of the 50 models without license (28%). This showcases promise in the use of GitHub as a platform for code release and maintenance, both because of ease of license upload and ease of public discourse. The models which did include a license varied in which open source license was used (Figure 2 and S1).

**Figure 2:**
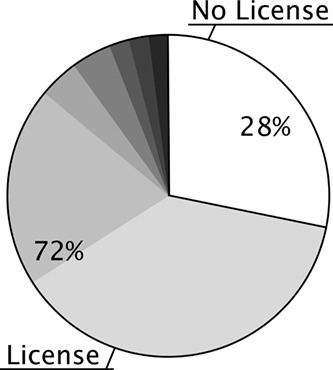
Licenses were not available for 28% of audited PLBAP models, making them unusable. A breakdown by license type is available in Figure S1.

#### Preprocessing, Training, and Inference Code Availability

Beyond licensing, the most obvious barrier to reproduction was the absence of code for one or more pipeline stages. Of the 50 models, 14 lacked preprocessing and featurization code entirely, 13 lacked training code, and 12 lacked inference (i.e., prediction) code. Interestingly, these gaps were not uniformly distributed: several pipelines provided training and inference scripts but omitted preprocessing scripts, presumably because well-known tools were used. In practice, this assumption frequently proved incorrect. For example, featurization workflows for CASF-2016 are sensitive to protonation state, hydrogen handling, cutoff distances, and more. As we discuss in later sections, we speculate that buggy or underspecified preprocessing code was the most common reason that runnable pipelines (those satisfying all five checklist items) failed to reproduce the reported PCC.

#### Pretrained or Finetuned Model Availability

Pretrained model weights represent perhaps the most practically consequential artifact for convenient, inference-only reproduction.^17^ They allow a researcher to bypass training entirely and directly verify reported scoring power or implement a given model for their own applications. Nevertheless, only 23 of 50 audited pipelines (46%) provided weights for the models which they report performances. For the remaining 27 pipelines, an independent reproduction attempt would require retraining from scratch, which is commonly infeasible given undocumented hyperparameters, training splits, or prohibitive compute costs. Even among pipelines that did provide weights, model weight files were occasionally ambiguous. In several cases, multiple weight files were present without documentation specifying which corresponds to the CASF-2016 evaluation. We assume selection of the wrong model file may be a recurring source of discrepancy in our reproduction attempts.

### Inconsistent Reproduction across Runnable Models

We define a pipeline as runnable if it satisfies all five checklist items. That is, it is licensed and provides preprocessing, training, and inference code, and provides pretrained weights. Of the 50 audited models, 17 met this definition. For each runnable pipeline, we executed the authors’ documented inference protocol on the CASF-2016 test set and computed the PCC. To obtain a confidence interval around our estimate, we applied nonparametric bootstrapping (*n* = 5000). Most published PLBAP models report only a single PCC value, with few reporting multiseed averages or bootstrapping results (Table S2). We consider the best reported PCC value when multiple are documented. We define a pipeline as *statistically reproducible* if the originally reported PCC falls within this 95% confidence interval (see Methods).

Of the 17 runnable pipelines, only nine were statistically reproducible, corresponding to a reproduction rate of 53% (Figure 3). The eight runnable, but nonreproducible pipelines exhibited failures attributable to three categories: (1) ambiguous pretrained weights or incomplete model release, (2) preprocessing and featurization bugs, and (3) broken or incompatible dependencies. Several models exhibited failures in more than one category. Failure profiles are detailed in Table 2 and elaborated on below.

**Figure 3:**
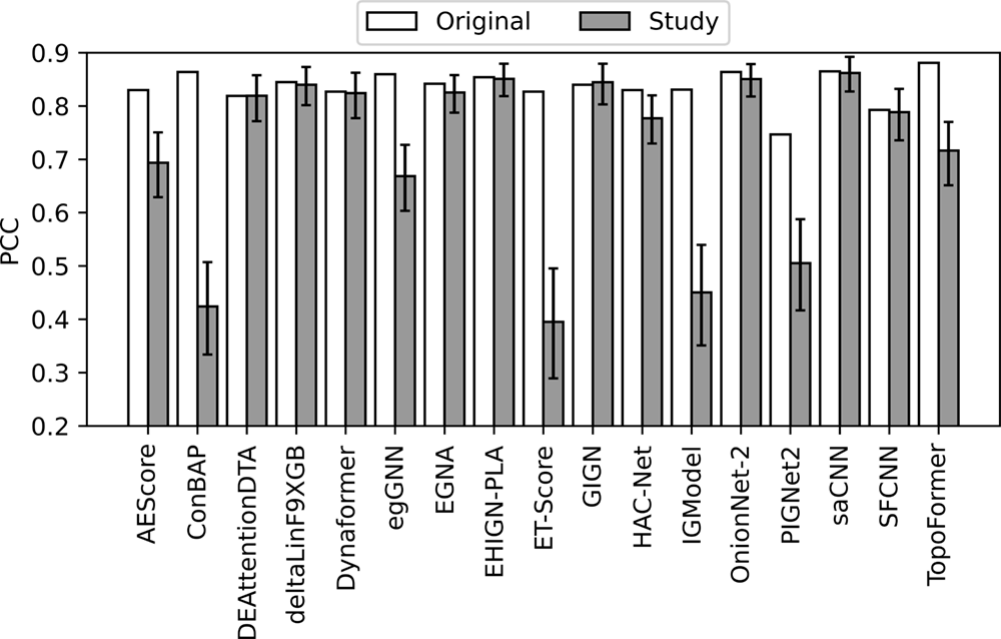
A comparison of performance as originally reported and attempted reproduction as Pearson’s correlation coefficient (PCC) for all runnable models. Of the 17 runnable models, nine were statistically reproducible within a 95% confidence interval. Bootstrapping (*n* = 5000) was performed to compute the 95% confidence interval, indicated with error bars.

**Table 2:**
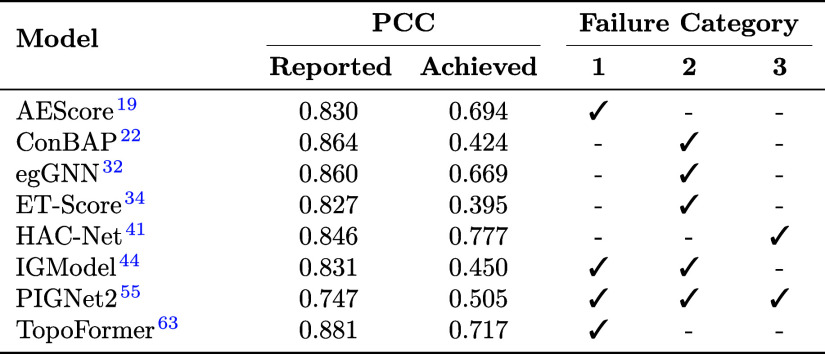
Failure Category Profiles for the Eight Runnable but Nonreproducible PLBAP Models^a^

a Achieved PCC is from the best reproduction attempt for each model. A model may appear in more than one category. Category definitions: (1) ambiguous pretrained weights or incomplete model release; (2) preprocessing and featurization bugs; (3) broken or incompatible dependencies.

#### Ambiguous Pretrained Weights and Incomplete Model Release

Four of the runnable, nonreproducible models provided multiple files containing pretrained model weights that were either ambiguous or incomplete (AEScore,^19^ IGModel,^44^ PIGNet2,^55^ TopoFormer^63^). In the case of AEScore, multiple checkpoint files were distributed across a Zenodo archive without clear documentation of which model corresponded to the reported best performance. We assume that incorrect checkpoint files were the primary source of discrepancy for AEScore, as no obvious bugs were otherwise identified. IGModel provided two model weight files (i.e., checkpoints) and PIGNet2 provided four; neither specified which checkpoint was used to obtain peak performance, but both models have compounding failure causes discussed below. For AEScore, IGModel, and PIGNet2, we ran the multiple models and present results for the best performing pretrained weight files (see Table S3). Topoformer reports the second-highest PCC of all 50 audited models (0.881) via a consensus of ten fine-tuned models. However, weights for only three of these ten models were made publicly available, and no explicit protocol was documented for aggregating predictions across fine-tuned checkpoints. Averaging the predictions over the three available models and yielded a PCC of 0.717.

#### Preprocessing and Featurization Bugs

Five models required modification of preprocessing and featurization code to be run to completion (ConBAP,^22^ egGNN,^32^ ET-Score,^34^ IGModel,^44^ PIGNet2 ^55^), with code alterations described in the commit logs of each repository’s study fork (see Table S1). After reasonable debugging attempts (2–4 h per model), inference produced a full set of predictions. ConBAP required fallback handling for RDKit^70^ valence errors encountered during pocket preprocessing. With our implemented solutions, ConBAP achieved a PCC of 0.424 against a reported 0.864, suggesting our RDKit fix altered downstream featurization and model performance substantially. Similarly, IGModel and PIGNet2 encountered multiple RDKit molecule parsing errors, which were compounded by one or more additional failure modes described. The egGNN pipeline lacked code for generating SMILES representations from CASF-2016 ligands. We employed the sdf_to_smi.py code from DEAttentionDTA^26^ to fill this gap, which resulted in smiles parsing errors and yielded a PCC of 0.669 against a reported 0.86. The ET-Score code for distance-based feature computation produced a divide-by-zero error which we ultimately resolved to achieve full runs for all complexes, achieving PCC of 0.385 versus the reported 0.827. It is possible that some of these errors are due to version mismatches of code packages used in preprocessing, which could be resolved with containerization of future PLBAP releases. We elaborate on this in the Future Proofing section.

#### Broken or Incompatible Dependencies

Environmental failures were related to the unsuccessful reproduction of two models (HAC-Net,^41^ PIGNet2 ^55^). HAC-Net’s preprocessing code relied on a deprecated, now broken API for the Atomic Charge Calculator II (ACC2).^71^ Though we attempted to replace the exact functionality with newer, functional ACC2 tools, we were unable to reproduce the original HAC-Net inference, achieving a PCC of 0.777 against reported 0.846. More complicated was PIGNet2, which required dependency updates from CUDA 11.1 to CUDA 12.x. Corresponding PyTorch^72^ and PyTorch Geometric version changes introduced API-breaking modifications to graph data structures, compounding the preprocessing errors described above.

One model, GIGN,^6^ achieved a PCC value slightly greater than that originally reported. This likely reflects minor differences in software version rather than a systematic error, supported by the fact that no functional modifications were made to the GIGN code beyond adding a feature to report the compute time per complex and modifying the paths to match our directory structure. This case was nonetheless counted as reproducible because the original reported value fell within our bootstrap confidence interval.

### Compute Time

To further characterize the practical usability of these pipelines, we recorded the wall-clock time required to featurize and score a single protein–ligand complex for each reproducible pipeline (Figure 4). Inference time spans from milliseconds for graph-transformer methods such as Dynaformer^9^ and DEAttentionDTA^26^ to hours for EGNA.^8^ Notably, there is no strong positive relationship between inference cost and reported accuracy. Several of the best performing models complete inference in under one second per complex. Compute time is, therefore, worth reporting alongside accuracy metrics in future PLBAP publications, as it directly determines whether a method is reasonably efficient for prospective screening campaigns.

**Figure 4:**
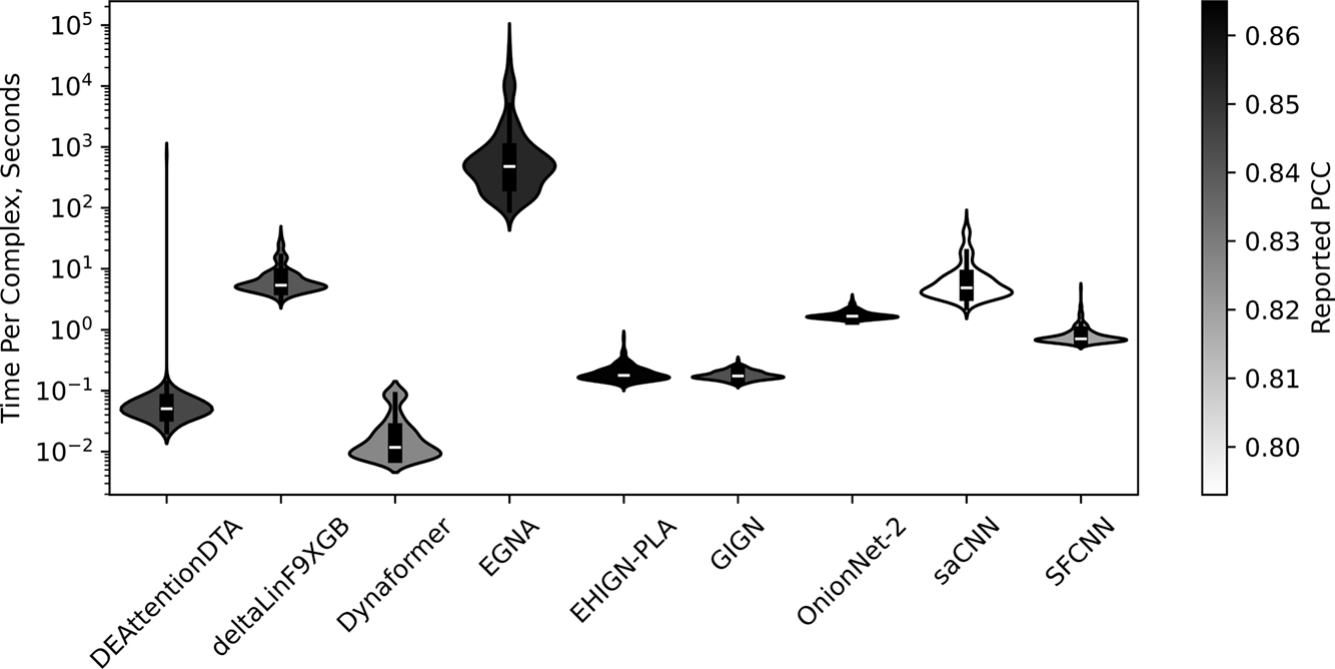
Per-complex wall times for combined preprocessing and inference varied across reproducible models, spanning from milliseconds to hours. Compute time did not correlate strongly with performance.

### Future Proofing and Convenience of Reproduction

The five-item checklist proposed here is intentionally minimal to prioritize its adoption. Authors who exceed this minimum stand to benefit directly. Pipelines that are easier to run and verify are more likely to be adopted as baselines, integrated into external workflows, and cited by other groups.^73,74^ Conversely, as we show here, pipelines that satisfy only the minimum checklist are vulnerable to the failure modes documented in this study. Several additional artifacts can meaningfully improve the depth and longevity of reproducibility. We discuss these here, both to justify their exclusion from our primary checklist and to offer forward-looking guidance for authors.

#### Environment Specifications and Containerization

A containerized compute environment, such as a Docker or Singularity image, captures the exact software stack used to generate published results, including operating system, CUDA version, and package versions.^75^ Containerization likely would have addressed the broken-dependency failures encountered in this study for HAC-Net and PIGNet2. At minimum, authors should provide a conda environment file with pinned package versions. Beyond this, authors are encouraged to provide a container image alongside their code repository.

#### Input Structures and Training Data

Raw protein–ligand complex structures used as input to PLBAP pipelines are not discussed here as a checklist item, and their absence from most published repositories is expected and appropriate. This is because all 50 audited models train and evaluate on PDBbind-derived structures, which are freely available to registered users directly from the PDBbind database^76^ but are not licensed for redistribution by third parties. That said, for models trained on other data sets, release of the input structures is highly recommended.

#### Train/Test Splits and Random Seeds

Train/test splits are commonly reported in PLBAP papers, and random seeds are often specified directly in published code, making these among the more accessible supplementary artifacts relative to others discussed here. Where splits are reported in prose rather than as a machine-readable file, we encourage authors to additionally provide a CSV or JSON of complex identifiers with split assignments. Similarly, where random seeds are embedded only within code, we advise authors to state their explicit definitions also within the published article or Supporting Information documents.

#### Intermediate Featurized Data

Intermediate featurized data (graphs, voxel grids, vectors) represent the output of preprocessing and the direct input to model training and inference. Sharing these files would allow a user to (1) verify that featurization of raw inputs is reproducible without inference, (2) bypass featurization entirely to verify inference performance with published pretrained models, and (3) verify that published training protocols reproduce published model weights. Where file size and licensing permit, we recommend sharing featurized training and test sets.

#### Per-Complex Prediction Results

Per-complex predicted affinities for the full test set are among the most useful, lowest-cost artifacts that an author can share. They allow independent verification of aggregate metrics such as PCC without rerunning the model at all. Moreover, they would allow fine-grained meta-analyses across pipelines. The per-complex predictions for each model able to be run in this study (17 total) are available at the respective study forks (see Table S1) to demonstrate this practice.

### Limitations

The CASF-2016 benchmark is the field’s established standard for scoring power evaluation, but it carries several limitations. First, the benchmark contains 285 complexes, a relatively small test set. Second, the reported PCC values across the 50 audited models span a narrow range (0.725–0.886). This reflects the maturity of the field on this particular benchmark, but also raises concerns about benchmark saturation. As more models are developed with CASF-2016 as an explicit target, the benchmark’s ability to discern between genuinely superior models and those optimized for the benchmark alone diminishes. Cross-benchmark validation can strengthen confidence in reported improvements and is encouraged as a complementary practice. Third, CASF-2016 uses experimental crystal structures as input, which represents an idealized setting relative to real-world virtual screening scenarios where docked or homology-modeled structures are more typical. Performance on crystal structures doesnot translate directly to practical utility, and reproducibility studies on docked-pose inputs are a valuable complement to the present work.^100^

Our reproduction protocol also may introduce limitations that should be considered when interpreting the reported failure rates. Namely, the reported reproduction results reflect the attempts of a single researcher who had no involvement with the development of any audited models. Some failures may reflect barriers that a more experienced user—in particular, the original authors—could resolve with additional context not available in the public documentation. We cannot exclude the possibility that some nonreproducible pipelines would yield better results under a more extended or collaborative reproduction protocol. All reproduction attempts were conducted on a single institutional high-performance computing cluster (Michigan State University HPCC) using two hardware partitions (H200 and V100 GPUs). Some dependency failures may be specific to these compute resources and could, perhaps, be resolved by running on the hardware or operating systems used by the original authors. Conversely, pipelines that succeeded on our hardware may encounter different failure modes elsewhere. We provide full environment specifications in study forks listed in Table S1.

Finally, our audit was conducted at a fixed point in time. Code repositories are living resources. Licenses were added to seven models following our public requests. Ongoing maintenance could change the runnability status of any pipeline after the time of writing. The checklist scores in Table 1 and the causes of failures in Table 2 reflect the state of each repository at the time of audit. We encourage readers to consult the original repositories directly for the current state of each pipeline.

## Methods

### Model Selection

Candidate models were identified through a systematic search of Google Scholar using the query protein ligand binding affinity CASF-2016 restricted to publications between January 2021 and December 2024. This date range was chosen to capture the most recent, state-of-the-art PLBAP pipelines. Models were included if they (1) reported a Pearson correlation coefficient (PCC) on the CASF-2016 scoring power benchmark, (2) predict binding affinity as p*K*, and (3) were structure-based, that is, they take three-dimensional protein–ligand complex coordinates as primary input. Sequence-based and ligand-only affinity prediction methods were excluded. This process yielded a final set of 50 models.

### Reproducibility Checklist

For each model, we assessed the public availability of five artifacts deemed necessary for independent reproduction of CASF-2016 inference: (1) an explicit software license to permit reuse, (2) preprocessing and featurization code, (3) training code, (4) inference code, and (5) pretrained or fine-tuned model weights. Each artifact was scored as present (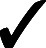) or absent (−) based on what was publicly available at the time of audit, reported in Table 1. We considered any information provided alongside the official publication, such as linked code or data repositories and supplementary materials. A model was classified as *runnable* if all five artifacts were present without requiring direct author contact or access to private resources.

### Reproduction Protocol

For each runnable model, we attempted to execute the author’s documented CASF-2016 inference pipeline using only publicly available code, documentation, and pretrained weights. We began each attempt from the CASF-2016 data set downloaded directly from PDBbind.^76^ The duration of researcher time actively spent on environment setup and dependency resolution, featurization, and inference (not including wall time for compute) was recorded and is documented in Table S4. Attempts that did not produce a valid set of CASF-2016 predictions due to missing code or data components were recorded as “Not attempted” and the primary barrier to completion was documented.

Where necessary to achieve a runnable state on our hardware, minor and explicitly documented modifications were permitted. Outside of error-handling, these changes were limited to adding command-line arguments to allow configurable file paths (replacing hard-coding), adding batch submission scripts for the high-performance computing cluster (HPCC), and adding conda environment specifications and/or install scripts and documentation. Code modifications related to error-handling were largely limited to (1) making code compatible with modern dependency versions (mostly PyTorch^72^), (2) resolving ligand parsing errors from RDKit^70^ or OpenBabel,^77^ and (3) resolving protein-related errors from OpenBabel. All changes are documented in model-specific forked repositories hosted on GitHub (see Table S1), with commit-level change summaries provided for each model in each repository. No modifications were made to model architecture, loss functions, or pretrained weights.

### Compute Environment

All reproduction attempts were conducted on the Michigan State University HPCC. Nodes were provisioned from either the amd20-v100 or amd24-h200 partitions, selected based on availability and each model’s hardware requirement, when stated. A subset of models that did not require GPU acceleration were executed on CPU-only allocations. Each model was run in an isolated conda environment. Python versions ranged from 2.7 to 3.12 across the audited pipelines, reflecting the heterogeneity of the dependency stacks encountered. The specific Python version and conda environment file used for each runnable model are provided in the forked repositories.

### Statistical Reproducibility Assessment

Before describing our assessment approach, we distinguish three related but distinct concepts that are easily conflated in reproducibility studies. *Practical rerunnability* refers to whether a pipeline can be executed at all by an independent researcher using only publicly available resources — the question addressed by the checklist audit. *Exact reproducibility* refers to whether an independent execution produces numerically identical results to those originally reported. *Statistical reproducibility*, the standard we adopt here, refers to whether an independently achieved result is consistent with the originally reported value within the uncertainty expected from finite sample estimation.

Exact reproducibility is essentially unattainable for modern deep learning pipelines. GPU floating-point operations are nondeterministic across hardware generations,^78^ cuDNN implementations vary across CUDA versions, and minor API differences between dependency versions can propagate to small numerical differences in outputs.^2^ Requiring exact agreement would therefore penalize pipelines for reasons unrelated to the validity of their reported results. Statistical reproducibility via bootstrap confidence intervals provides a principled and practically meaningful alternative: it asks not whether we obtained the identical number, but whether the originally reported value is consistent with what an independent execution of the same pipeline produces on the same data.

For each model that successfully completed inference, we computed the PCC between predicted and experimentally measured binding affinities (p*K*) across the 285 CASF-2016 test complexes. To obtain a confidence interval, we applied nonparametric bootstrap resampling with 5,000 iterations. The 2.5th and 97.5th percentiles of this distribution were taken as the bounds of a 95% confidence interval. A model was classified as *statistically reproducible* if the originally reported PCC fell within this interval. For models where multiple model weight files were available without documentation specifying which was used in the original evaluation, we ran inference with each pretrained model and report results for the best. The specific weight file used for each model is documented in Table S3.

## Conclusion

We conducted a systematic reproducibility study of 50 PLBAP models benchmarked on CASF-2016 which included the audit of five reproducibility-critical artifacts: a license, code for preprocessing, training, and inference, and model weights. Only 17 pipelines satisfied this checklist. Of these 17 models, only nine reproduced the originally reported PCC within a bootstrapped 95% confidence interval. The most common barriers to reproduction were missing or ambiguous pretrained weights and broken preprocessing code. The latter of these contributed multiple reproduction failures even when all five artifacts were present. These findings motivate scrutiny against cross-study comparisons that cite previously reported CASF-2016 PCC values without independent verification.

We propose the five-item checklist introduced here as a lightweight community standard to encourage the publication of resources for reproduction alongside PLBAP papers. Providing a license, complete code for each pipeline stage, and clear pretrained model weights at the time of publication are each individually achievable. Together, though, they represent only the minimum necessary for published PLBAP results to be independently verifiable. We hope that journals, reviewers, and authors in this space will adopt this checklist, or one of a similar sentiment, as a routine part of the publication process, so that future benchmarking claims can be built on a more reproducible foundation.

## Supplementary Material



## Data Availability

Per complex p*K* prediction and timing results for each model, plotting scripts, metadata, and links to forked repositories of rerun models are available at https://github.com/WoldringLabMSU/PLBAP_Reproducibility.

## Notes

Code repository links and additional figures and tables as mentioned in the main text (PDF)

## Abbreviations

PLBAP: Protein–ligand binding affinity prediction
PCC: Pearson correlation coefficient
