# Supplementary material for: Critical Artifacts Improve Reproducibility of Protein–Ligand Binding Affinity Prediction Models on CASF-2016
Source: J Chem Inf Model. 2026 Jun 24;66(13):7453–61. doi: 10.1021/acs.jcim.6c01192 (PMC13370855; doi:10.1021/acs.jcim.6c01192)
Supplement: Supplementary file 1 [file ci6c01192_si_001.pdf]

# Supplemental Information: Critical Artifacts Improve Reproducibility of Protein-Ligand Binding Affinity Prediction Models on CASF-2016

Joelle N. Eaves[1,2], Angeline A. Needs[1,2], and Daniel R. Woldring*[1,2]

[1]Department of Chemical Engineering and Materials Science, Michigan State University, East Lansing, MI 48824, USA
[2]Institute for Quantitative Health Science and Engineering, Michigan State University, East Lansing, MI 48824, USA

*Email: woldring@msu.edu

## Code Repositories

We investigated 50 PLBAP models published between 2021 and 2024. Table S1 documents the original code repositories, if available, for each pipeline. For models which a reproduction attempt was able to be made, links to our study forks are also provided.

## PLBAP Model Metadata

The main GitHub repository for this work is https://github.com/WoldringLabMSU/PLBAP_Reproducibility. There, json files following a common schema document metadata for each model and reproduction attempt including python version, conda environment used, model checkpoint file used (see also Table S3), license type, last commit date, failure causes, and more.

## License Availability

For each model, we audited the type of license available, if any. Results in Figure S1 show that 14/50 models (28%) had no license at all, prohibiting their reuse and excluding them from our investigation. Of the 72% of models that did provide licenses, 19 models used MIT license, 10 used GPL-3.0, and the remainder used other license types.

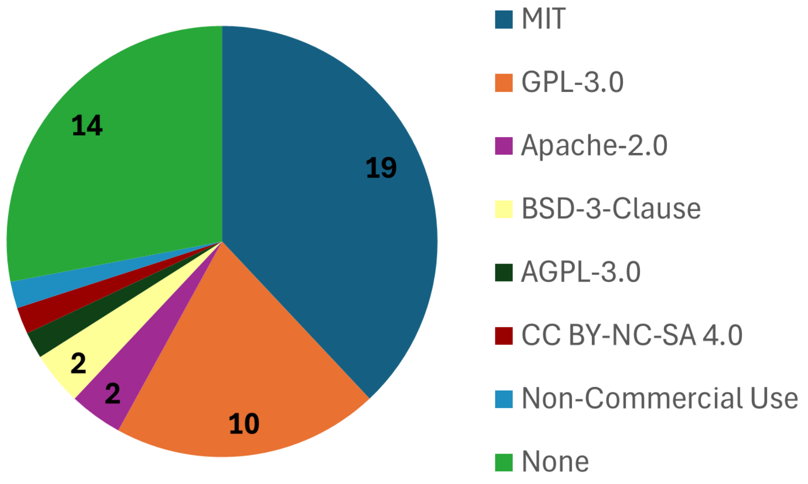


Figure S1: Licenses were not available for 28% of audited PLBAP models, making them entirely unusable. Of the 72% of models which did provide a license, the most common types were MIT and GPL-3.0 licenses.

Table S1: Original repositories for each audited PLBAP pipeline, if available. For models with all five checklist items, reproduction attempts were made using code available at "Study Fork" links.

| Model | Original Repository | Study Fork |
|---|---|---|
| AEScore [1] | RMeli/aescore | jeavesj/aescore |
| BAPA [2] | Blue1993/BAPA | - |
| CGraphDTA [3] | - | - |
| ConBAP [4] | ld139/ConBAP | - |
| CPIScore [5] | liang2508/CPIScore | - |
| CurvAGN [6] | tumancao/CurvAGN | - |
| DAAP [7] | https://gitlab.com/mahnewton/daap | - |
| DEAttentionDTA [8] | whatamazing1/DEAttentionDTA | jeavesj/DEAttentionDTA |
| DeepBindGCN [9] | haiping1010/DeepBindGCN | - |
| DeepTGIN [10] | zhc-moushang/DeepTGIN | - |
| delta_LinF9_XGB [11] | cyangNYU/delta_LinF9_XGB | jeavesj/delta_LinF9_XGB |
| Dowker [12] | LiuXiangMath/Dowker-Complex-Based-ML | - |
| Dynaformer [13] | Minys233/Dynaformer | jeavesj/Dynaformer |
| ECIF [14] | DIFACQUIM/ECIF | - |
| egGNN [15] | xfcui/egGNN | jeavesj/egGNN |
| EGNA [16] | chunqiux/EGNA | jeavesj/EGNA |
| EHIGN-PLA [17] | guaguabujianle/EHIGN_PLA | jeavesj/EHIGN_PLA |
| EISA-Score [18] | MathIntelligence/EISA-Score | - |
| ET-Score [19] | miladrayka/ET_Score | jeavesj/ET_Score |
| FGNN [20] | LinaDongXMU/FGNN | - |
| GB-Score [21] | miladrayka/GB_Score | - |
| GCNN01 [22] | IanYMY/GCNN | - |
| GGL-Score [23] | MathIntelligence/GGL-ETA-Score | - |
| GIaNt [24] | PaddlePaddle/PaddleHelix/tree/dev/apps/drug_target_interaction/giant | - |
| GIGN [25] | guaguabujianle/GIGN | jeavesj/GIGN |
| GraphScoreDTA [26] | CSUBioGroup/GraphscoreDTA | - |
| HAC-Net [27] | gregory-kyro/HAC-Net | jeavesj/HAC-Net |
| HaPPy [28] | Jthy-af/HaPPy | - |
| HydraScreen [29] | Ro5-ai/hydrascreen | - |
| IGModel [30] | zchwang/IGModel | jeavesj/IGModel |
| IMCPiDB-Score [31] | debbydanwang/BAP | - |
| LGN [32] | gojx1998/LGN | - |

*Continued on next page*

| | | |
|---|---|---|
| LigityScore[33] | https://gitlab.com/josephazzopardi/ligityscore | - |
| MFE[34] | Sultans0fSwing/MFE | - |
| MM-DRPNet[35] | Bigrock-dd/MMDRPv1 | - |
| MP-GNN[36] | Alibaba-DAMO-DrugAI/MGNN | - |
| MSECIF[37] | koji11235/MSECIFv2 | - |
| MSSDTA[38] | - | - |
| OnionNet-2[39] | zchwang/OnionNet-2 | jeavesj/OnionNet-2 |
| P3-Score[40] | lichuang0/P3-Score | |
| PerSpect ML[41] | fdmm1989/PersistentHodgeLaplacian | |
| PIGNet2[42] | ACE-KAIST/PIGNet2 | jeavesj/PIGNet2 |
| PLANET[43] | ComputArtCMCG/PLANET | - |
| pointnet[44] | wyji001/Point-Cloud | - |
| PPS-ML[45] | LiuRanMath/Persistent-path-spectral-based-ML | - |
| RFL-Score[46] | rflscore/rfl-score_v1 | - |
| saCNN[47] | xfcui/saCNN | jeavesj/saCNN |
| SFCNN[48] | bioinfocqupt/Sfcnn | jeavesj/Sfcnn |
| SMPLIP-Score[49] | college-of-pharmacy-gachon-university/SMPLIP-Score | - |
| TopoFormer[50] | WeilabMSU/TopoFormer | - |

Unless specified, all repositories are hosted on GitHub and links begin with https://github.com/.

Table S2: Models reporting statistical information in addition to a single PCC value.

| Model | Reported PCC Information | Method |
|---|---|---|
| AEScore [1] | $[0.76, 0.83]_{CI90\%}$ | Bootstrapping, n=10,000 |
| ConBAP [4] | 0.864 (0.005) | mean (standard deviation), n=3 |
| EHIGN-PLA [17] | 0.854 (0.004) | mean (standard deviation), n=3 |
| ET-Score [19] | 0.827 (0.003) | mean (standard deviation, n=3 |
| GIGN [25] | 0.840 (0.007) | mean (standard deviation), n=3 |

Table S3: Link to model weight checkpoint used for each model we attempted to reproduce.

| **Model** | **Weight Checkpoint File Link** |
|---|---|
| AEScore[1] | https://doi.org/10.5281/zenodo.4155365 (model: experiments/CASF-2016-consensus-nohetatm-PH/out) |
| ConBAP[4] | https://github.com/jeavesj/ConBAP/blob/main/supervised/model/20231007_111336_ConBAP_repeat0/model/epoch-292%2C%20train_loss-0.1220%2C%20train_rmse-0.3493%2C%20valid_rmse-1.1663%2C%20valid_pr-0.7788.pt |
| DEAttentionDTA[8] | https://github.com/jeavesj/DEAttentionDTA/tree/main/src/model |
| delta_LinF9_XGB[11] | https://github.com/jeavesj/delta_LinF9_XGB/tree/main/saved_model |
| Dynaformer[13] | https://1drv.ms/f/s!Ah9r82oejjV8n0Bw4vAddMLBK-m-?e=95ciZv |
| egGNN[15] | https://github.com/jeavesj/egGNN/blob/master/egGNN/checkpoint/16-8596.sav |
| EGNA[16] | https://github.com/jeavesj/EGNA/tree/main/models |
| EHIGN-PLA[17] | https://github.com/jeavesj/EHIGN_PLA/blob/main/model/20230120_135757_EHIGN_repeat0/model/epoch-144%2C%20train_loss-0.5772%2C%20train_rmse-0.7598%2C%20valid_rmse-1.1799%2C%20valid_pr-0.7718.pt |
| ET-Score[19] | https://figshare.com/articles/software/ET-Score/23684538 |
| GIGN[25] | https://github.com/jeavesj/GIGN/blob/main/GIGN/model/20221121_074758_GIGN_repeat0/model/epoch-532%2C%20train_loss-0.1162%2C%20train_rmse-0.3408%2C%20valid_rmse-1.1564%2C%20valid_pr-0.7813.pt |
| HAC-Net[27] | https://github.com/gregory-kyro/HAC-Net/tree/main/HACNet/parameter_files |
| IGModel[30] | https://github.com/jeavesj/IGModel/blob/master/models/saved_model.pth |
| OnionNet-2[39] | https://zenodo.org/records/10728089 |
| PIGNet2[42] | https://github.com/jeavesj/PIGNet2/blob/main/src/ckpt/pda_0.pt |
| saCNN[47] | https://github.com/jeavesj/saCNN/blob/main/checkpoint/model.pkl |
| Sfcnn[48] | https://github.com/jeavesj/Sfcnn/blob/main/weights_22_112-0.0083.h5 |
| TopoFormer[50] | https://weilab.math.msu.edu/Downloads/TopoFormer/finetuned_casf2016_3models.tar.gz |

Table S4: Active researcher time spent per-model on setup, dependency resolution, and debugging. This excludes model run time.

| Model | Time Range (hours) |
|---|---|
| AEScore [1] | 2-4 |
| ConBAP [4] | 2-4 |
| DEAttention [8] | 2-4 |
| delta_LinF9_XGB [11] | 2-4 |
| Dynaformer [13] | 4-6 |
| egGNN [15] | 2-4 |
| EGNA [16] | 2-4 |
| EHIGN-PLA [17] | 2-4 |
| ET-Score [19] | 2-4 |
| GIGN [25] | <2 |
| HAC-Net [27] | 8-12 |
| IGModel [30] | 2-4 |
| OnionNet-2 [39] | 2-4 |
| PIGNet2 [42] | 2-4 |
| saCNN [47] | 4-6 |
| SFCNN [48] | <2 |
| TopoFormer [50] | 6-8 |

# References


[1] Meli, R.; Anighoro, A.; Bodkin, M. J.; Morris, G. M.; Biggin, P. C. Learning protein-ligand binding affinity with atomic environment vectors. *Journal of Cheminformatics* **2021**, *13*, 59.

[2] Seo, S.; Choi, J.; Park, S.; Ahn, J. Binding affinity prediction for protein–ligand complex using deep attention mechanism based on intermolecular interactions. *BMC bioinformatics* **2021**, *22*, 542.

[3] Wang, K.; Li, M. Fusion-Based Deep Learning Architecture for Detecting Drug-Target Binding Affinity Using Target and Drug Sequence and Structure. *IEEE Journal of Biomedical and Health Informatics* **2023**, *27*, 6112–6120.

[4] Luo, D.; Liu, D.; Qu, X.; Dong, L.; Wang, B. Enhancing Generalizability in Protein–Ligand Binding Affinity Prediction with Multimodal Contrastive Learning. *Journal of Chemical Information and Modeling* **2024**, *64*, 1892–1906, PMID: 38441880.

[5] Liang, L.; Duan, Y.; Zeng, C.; Wan, B.; Yao, H.; Liu, H.; Lu, T.; Zhang, Y.; Chen, Y.; Shen, J. CPIScore: A Deep Learning Approach for Rapid Scoring and Interpretation of Protein–Ligand Binding Interactions. *Journal of Chemical Information and Modeling* **2024**, *64*, 8809–8823, PMID: 39563077.

[6] Wu, J.; Chen, H.; Cheng, M.; Xiong, H. Curvagn: curvature-based adaptive graph neural networks for predicting protein-ligand binding affinity. *BMC bioinformatics* **2023**, *24*, 378.

[7] Rahman, J.; Newton, M. H.; Ali, M. E.; Sattar, A. Distance plus attention for binding affinity prediction. *Journal of Cheminformatics* **2024**, *16*, 52.

[8] Chen, X.; Huang, J.; Shen, T.; Zhang, H.; Xu, L.; Yang, M.; Xie, X.; Yan, Y.; Yan, J. DEAttentionDTA: protein–ligand binding affinity prediction based on dynamic embedding and self-attention. *Bioinformatics* **2024**, *40*, btae319.

[9] Zhang, H.; Saravanan, K. M.; Zhang, J. Z. H. DeepBindGCN: Integrating Molecular Vector Representation with Graph Convolutional Neural Networks for Protein–Ligand Interaction Prediction. *Molecules* **2023**, *28*.

[10] Wang, G.; Zhang, H.; Shao, M.; Feng, Y.; Cao, C.; Hu, X. DeepTGIN: a novel hybrid multimodal approach using transformers and graph isomorphism networks for protein-ligand binding affinity prediction. *Journal of Cheminformatics* **2024**, *16*, 147.

[11] Yang, C.; Zhang, Y. Delta Machine Learning to Improve Scoring-Ranking-Screening Performances of Protein–Ligand Scoring Functions. *Journal of Chemical Information and Modeling* **2022**, *62*, 2696–2712, PMID: 35579568.

[12] Liu, X.; Feng, H.; Wu, J.; Xia, K. Dowker complex based machine learning (DCML) models for protein-ligand binding affinity prediction. *PLoS computational biology* **2022**, *18*, e1009943.

[13] Min, Y.; Wei, Y.; Wang, P.; Wang, X.; Li, H.; Wu, N.; Bauer, S.; Zheng, S.; Shi, Y.; Wang, Y.; others From Static to Dynamic Structures: Improving Binding Affinity Prediction with Graph-Based Deep Learning. *Advanced Science* **2024**, *11*, 2405404.

[14] Sánchez-Cruz, N.; Medina-Franco, J. L.; Mestres, J.; Barril, X. Extended connectivity interaction features: improving binding affinity prediction through chemical description. *Bioinformatics* **2020**, *37*, 1376–1382.

[15] Jiao, Q.; Qiu, Z.; Wang, Y.; Chen, C.; Yang, Z.; Cui, X. Edge-Gated Graph Neural Network for Predicting Protein-Ligand Binding Affinities. 2021 IEEE International Conference on Bioinformatics and Biomedicine (BIBM). 2021; pp 334–339.

[16] Xia, C.; Feng, S.-H.; Xia, Y.; Pan, X.; Shen, H.-B. Leveraging scaffold information to predict protein–ligand binding affinity with an empirical graph neural network. *Briefings in bioinformatics* **2023**, *24*, bbac603.

[17] Yang, Z.; Zhong, W.; Lv, Q.; Dong, T.; Chen, G.; Chen, C. Y.-C. Interaction-based inductive bias in graph neural networks: enhancing protein-ligand binding affinity predictions from 3d structures. *IEEE Transactions on Pattern Analysis and Machine Intelligence* **2024**, *46*, 8191–8208.

[18] Rana, M. M.; Nguyen, D. D. EISA-Score: Element Interactive Surface Area Score for Protein–Ligand Binding Affinity Prediction. *Journal of Chemical Information and Modeling* **2022**, *62*, 4329–4341, PMID: 36108270.

[19] Rayka, M.; Karimi-Jafari, M. H.; Firouzi, R. ET-score: Improving Protein-ligand Binding Affinity Prediction Based on Distance-weighted Interatomic Contact Features Using Extremely Randomized Trees Algorithm. *Molecular Informatics* **2021**, *40*, 2060084.

[20] Dong, L.; Shi, S.; Qu, X.; Luo, D.; Wang, B. Ligand binding affinity prediction with fusion of graph neural networks and 3D structure-based complex graph. *Physical Chemistry Chemical Physics* **2023**, *25*, 24110–24120.

[21] Rayka, M.; Firouzi, R. GB-score: Minimally designed machine learning scoring function based on distance-weighted interatomic contact features. *Molecular Informatics* **2023**, *42*, 2200135.

[22] Yang, Y.; Zhang, R.; Lin, Z. Enhancing protein-ligand binding affinity prediction through sequential fusion of graph and convolutional neural networks. *Journal of Computational Chemistry* **2024**, *45*, 2929–2940.

[23] Rana, M. M.; Nguyen, D. D. Geometric graph learning with extended atom-types features for protein-ligand binding affinity prediction. *Computers in Biology and Medicine* **2023**, *164*, 107250.

[24] Li, S.; Zhou, J.; Xu, T.; Huang, L.; Wang, F.; Xiong, H.; Huang, W.; Dou, D.; Xiong, H. Giant: Protein-ligand binding affinity prediction via geometry-aware interactive graph neural network. *IEEE Transactions on Knowledge and Data Engineering* **2023**, *36*, 1991–2008.

[25] Yang, Z.; Zhong, W.; Lv, Q.; Dong, T.; Yu-Chian Chen, C. Geometric interaction graph neural network for predicting protein–ligand binding affinities from 3d structures (gign). *The journal of physical chemistry letters* **2023**, *14*, 2020–2033.

[26] Wang, K.; Zhou, R.; Tang, J.; Li, M. GraphscoreDTA: optimized graph neural network for protein–ligand binding affinity prediction. *Bioinformatics* **2023**, *39*, btad340.

[27] Kyro, G. W.; Brent, R. I.; Batista, V. S. Hac-net: A hybrid attention-based convolutional neural network for highly accurate protein–ligand binding affinity prediction. *Journal of Chemical Information and Modeling* **2023**, *63*, 1947–1960.

[28] Zhang, X.; Li, Y.; Wang, J.; Xu, G.; Gu, Y. A multi-perspective model for protein–ligand-binding affinity prediction. *Interdisciplinary Sciences: Computational Life Sciences* **2023**, *15*, 696–709.

[29] Prat, A.; Abdel Aty, H.; Bastas, O.; Kamuntavičius, G.; Paquet, T.; Norvaišas, P.; Gasparotto, P.; Tal, R. HydraScreen: A Generalizable Structure-Based Deep Learning Approach to Drug Discovery. *Journal of Chemical Information and Modeling* **2024**, *64*, 5817–5831, PMID: 39037942.

[30] Wang, Z.; Wang, S.; Li, Y.; Guo, J.; Wei, Y.; Mu, Y.; Zheng, L.; Li, W. A new paradigm for applying deep learning to protein–ligand interaction prediction. *Briefings in Bioinformatics* **2024**, *25*, bbae145.

[31] Wang, D. D.; Chan, M.-T. Protein-ligand binding affinity prediction based on profiles of intermolecular contacts. *Computational and Structural Biotechnology Journal* **2022**, *20*, 1088–1096.

[32] Guo, J. Improving structure-based protein-ligand affinity prediction by graph representation learning and ensemble learning. *PLoS One* **2024**, *19*, e0296676.

[33] Azzopardi, J.; Ebejer, J. P. LigityScore: A CNN-Based Method for Binding Affinity Predictions. Biomedical Engineering Systems and Technologies. Cham, 2022; pp 18–44.

[34] Xu, S.; Shen, L.; Zhang, M.; Jiang, C.; Zhang, X.; Xu, Y.; Liu, J.; Liu, X. Surface-based multimodal protein–ligand binding affinity prediction. *Bioinformatics* **2024**, *40*, btae413.

[35] Liu, D.; Song, T.; Wang, S. MM-DRPNet: A multimodal dynamic radial partitioning network for enhanced protein–ligand binding affinity prediction. *Computational and Structural Biotechnology Journal* **2024**, *23*, 4396–4405.

[36] Li, M.; Cao, Y.; Liu, X.; Ji, H. Structure-aware graph attention diffusion network for protein–ligand binding affinity prediction. *IEEE Transactions on Neural Networks and Learning Systems* **2023**, *35*, 18370–18380.

[37] Shiota, K.; Akutsu, T. Multi-shelled ECIF: improved extended connectivity interaction features for accurate binding affinity prediction. *Bioinformatics Advances* **2023**, *3*, vbad155.

[38] Wang, H.; Wang, S.; Ouyang, X.; Zhao, J.; He, Z.; Gao, T. Predicting Protein-Ligand Binding Affinity with Multi-Scale Structural Features. 2023 IEEE International Conference on Bioinformatics and Biomedicine (BIBM). 2023; pp 63–68.

[39] Wang, Z.; Zheng, L.; Liu, Y.; Qu, Y.; Li, Y.-Q.; Zhao, M.; Mu, Y.; Li, W. OnionNet-2: a convolutional neural network model for predicting protein-ligand binding affinity based on residue-atom contacting shells. *Frontiers in chemistry* **2021**, *9*, 753002.

[40] Li, C.; Zhang, A.; Wang, L.; Zuo, J.; Zhu, C.; Xu, J.; Wang, M.; Zhang, J. Z. Development of a polynomial scoring function P3-Score for improved scoring and ranking powers. *Chemical Physics Letters* **2023**, *824*, 140547.

[41] Meng, Z.; Xia, K. Persistent spectral–based machine learning (PerSpect ML) for protein-ligand binding affinity prediction. *Science advances* **2021**, *7*, eabc5329.

[42] Moon, S.; Hwang, S.-Y.; Lim, J.; Kim, W. Y. PIGNet2: a versatile deep learning-based protein–ligand interaction prediction model for binding affinity scoring and virtual screening. *Digital Discovery* **2024**, *3*, 287–299.

[43] Zhang, X.; Gao, H.; Wang, H.; Chen, Z.; Zhang, Z.; Chen, X.; Li, Y.; Qi, Y.; Wang, R. Planet: a multi-objective graph neural network model for protein–ligand binding affinity prediction. *Journal of chemical information and modeling* **2023**, *64*, 2205–2220.

[44] Wang, Y.; Wu, S.; Duan, Y.; Huang, Y. A point cloud-based deep learning strategy for protein–ligand binding affinity prediction. *Briefings in bioinformatics* **2022**, *23*, bbab474.

[45] Liu, R.; Liu, X.; Wu, J. Persistent Path-Spectral (PPS) Based Machine Learning for Protein–Ligand Binding Affinity Prediction. *Journal of Chemical Information and Modeling* **2023**, *63*, 1066–1075, PMID: 36647267.

[46] Arrua, O. E.; Aderhold, A.; Werhli, A. V.; Dos Santos Machado, K. RFL-Score: Random Forest with Lasso Scoring Function for Protein-Ligand Molecular Docking. 2024 IEEE Conference on Computational Intelligence in Bioinformatics and Computational Biology (CIBCB). 2024; pp 1–8.

[47] Wang, Y.; Qiu, Z.; Jiao, Q.; Chen, C.; Meng, Z.; Cui, X. Structure-Based Protein-Drug Affinity Prediction with Spatial Attention Mechanisms. 2021 IEEE International Conference on Bioinformatics and Biomedicine (BIBM). 2021; pp 92–97.

[48] Wang, Y.; Wei, Z.; Xi, L. Sfcnn: a novel scoring function based on 3D convolutional neural network for accurate and stable protein–ligand affinity prediction. *BMC bioinformatics* **2022**, *23*, 222.

[49] Kumar, S.; Kim, M.-h. SMPLIP-Score: predicting ligand binding affinity from simple and interpretable on-the-fly interaction fingerprint pattern descriptors. *Journal of cheminformatics* **2021**, *13*, 28.

[50] Chen, D.; Liu, J.; Wei, G.-W. Multiscale topology-enabled structure-to-sequence transformer for protein–ligand interaction predictions. *Nature Machine Intelligence* **2024**, *6*, 799–810.
